# *Lactiplantibacillus plantarum* P101 Attenuated Cyclophosphamide-Induced Liver Injury in Mice by Regulating the Nrf2/ARE Signaling Pathway

**DOI:** 10.3390/ijms241713424

**Published:** 2023-08-30

**Authors:** Tao You, Yu Zhao, Shanji Liu, Hengyi Xu

**Affiliations:** State Key Laboratory of Food Science and Resources, Nanchang University, 235 Nanjing East Road, Nanchang 330047, China; 407900210112@email.ncu.edu.cn (T.Y.); 5603515014@email.ncu.edu.cn (Y.Z.); 412314919032@email.ncu.edu.cn (S.L.)

**Keywords:** cyclophosphamide, *Lactiplantibacillus plantarum* P101, liver injury, oxidative stress

## Abstract

Cyclophosphamide causes side effects in cancer patients, including hepatotoxicity. Probiotics have recently emerged as potential approaches for the administration of many diseases. This study aimed to evaluate the protective effects of *Lactiplantibacillus plantarum* P101 against cyclophosphamide-induced liver injury and elucidate the underlying mechanism. In this study, *Lactiplantibacillus plantarum* P101 or *Lactobacillus rhamnosus* GG were pre-administered to mice with varying duration (1 week, 2 weeks, and 3 weeks) before being intraperitoneally injected with cyclophosphamide at a dose of 30 mg/kg/day for 7 days to induce liver injury. Results demonstrated that cyclophosphamide-induced liver injury was characterized by histopathological disorders, including irregular central venous shape and hepatic vascular rupture, as well as a severe inflammation response and oxidative stress. The administration of probiotics for 3 weeks exerted the most significant improvements in alleviating liver injury, oxidative stress, and inflammation when compared to the shorter intervention duration. Notably, *Lactiplantibacillus plantarum* P101 exhibited more pronounced effects than *Lactobacillus rhamnosus* GG. Furthermore, *Lactiplantibacillus plantarum* P101 enhanced the antioxidant defense system by activating the Nrf2/ARE signaling pathway, ultimately alleviating hepatotoxicity and hepatocyte apoptosis. In conclusion, this study highlighted the potential of *Lactiplantibacillus plantarum* P101 to alleviate cyclophosphamide-induced hepatotoxicity.

## 1. Introduction

Cyclophosphamide (CTX) is an alkylating compound used as a broad-spectrum anticancer agent in clinical chemotherapy [1]. Despite its effectiveness in killing tumor cells, the non-targeted and non-specific properties of CTX might lead to normal cell damage and induce adverse side effects, including immunosuppression, reproductive toxicity, and hepatotoxicity [2,3]. Those side effects have affected millions of cancer patients around the world, imposing significant financial and health burdens on them. Upon metabolism in organisms, the CTX is converted to the phosphamides mustard and acrolein by the cytochrome P450 enzymes (CYP450), which predominantly present in hepatocytes [4,5]. Previous studies have suggested that CTX-induced liver injury is associated with acrolein [6]. Specifically, acrolein could induce reactive oxygen species (ROS) generation and accumulation in the liver, followed by the emergence of oxidative stress. That will lead to structural damage to the membranes of various organelles, including the mitochondrial membrane in hepatocytes, and ultimately severe hepatocyte inflammation and apoptosis [7,8].

The liver is the primary and most important organ involved in the metabolism of CTX [9]. Hepatotoxicity caused by CTX has been attributed to oxidative stress, which disrupts the redox balance of liver tissue and leads to the accumulation of ROS when administered at high doses or with continuous accumulation [10]. Additionally, CTX has been demonstrated to downregulate the transcription of nuclear factor erythroid 2-related factor 2 (Nrf2), which is a key component of the antioxidant system of the liver [7]. Li et al. [4] demonstrated that pyrroloquinoline quinone could alleviate CTX-induced hepatotoxicity by upregulating the Nrf2/ARE pathway, indicating the potential functions of antioxidant therapy against CTX-induced hepatotoxicity. However, traditional liver-protective drugs used to treat chemotherapy drug damage have difficulty controlling the dose and could even exacerbate liver damage. Additionally, natural plant extracts, such as naringin [11], plant alcohol extract [12,13], and punicalagin [6], have been reported to have free radical scavenging activity and anti-inflammation properties, but their application is limited by issues such as difficulty in extraction, high cost, and dosage. Therefore, there is a pressing need for safe and sustainable treatments to mitigate the adverse effects of chemotherapy drugs. Recent evidence has strongly indicated a close relationship between gut microbes and the development of liver injury induced by various factors [14,15,16]. Additionally, the role of probiotics in regulating the structure of intestinal flora has been widely reported. Hence, utilizing probiotics to alleviate the side effects of chemotherapeutic drugs may be a potential effective therapy that requires further investigation.

Probiotics are known to confer beneficial and promoting effects on the host [17], including the modulation of gut microbiome composition and immune response, strengthening of the intestinal barrier, and antioxidant activity [18,19], and they are safe and reliable [20]. In addition, the outstanding antioxidative property is another critical feature of probiotics [21]. Numerous studies have suggested that probiotic strains can alleviate oxidative stress and inflammation in rats with nonalcoholic fatty liver disease [22,23,24]. Gao et al. [25] have found that the hepatic protective function of probiotics may be attributed to activating the Nrf2 signal pathway. Another common adverse reaction to antitumor therapy is gastrointestinal disorder, which can be improved by the administration of probiotic supplements [20]. That indicated that the probiotic may be a novel strategy against CTX-induced liver injury. Although clinical data regarding the direct use of probiotics to treat tumors is limited, several clinical trials have demonstrated the efficacy of probiotics in attenuating chemotherapy-induced adverse reactions. To optimize the efficacy of probiotic interventions, researchers have conducted numerous investigations and efforts into factors such as dose or strain selection, prebiotic and probiotic combination, and fecal bacteria transplantation. Despite these efforts, the precise mechanisms by which probiotics elicit their advantageous effects are not well comprehended, and additional research is required to determine whether the duration of supplementation is associated with the magnitude of these impacts.

In this study, the potential preventive effects of *L. plantarum* P101 and LGG on CTX-induced hepatic injury in mice were investigated. Specifically, two strains were orally pre-administered to mice for varying durations, and then mice were administrated CTX to model the effects. The work aimed to assess the protective effects of different strains and intervention durations on the liver, hoping to elucidate the responsible mechanism and provide new perspectives for probiotic application.

## 2. Results

### 2.1. L. plantarum P101 Pretreatment Reduced the Negative Effects of CTX Intervention on Organ Coefficients in Mice

**Figure 1:**
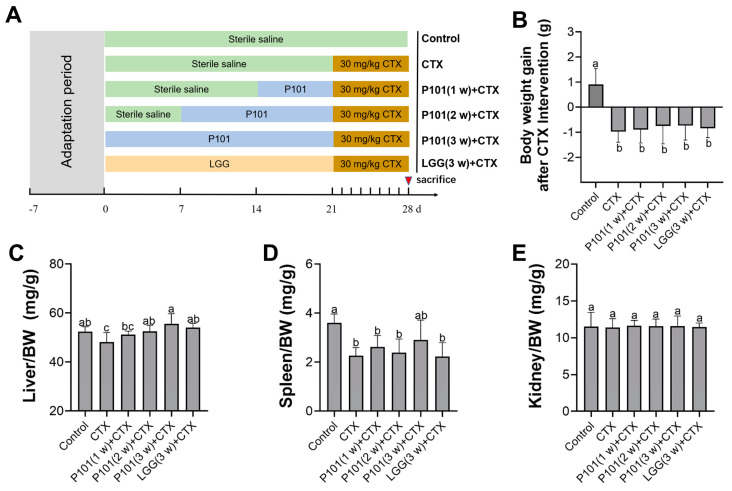
The diagram of the experimental design and the effects of CTX on organ indexes in mice. (**A**) Experimental groups and respective treatments. (**B**) Body weight gain after CTX intervention. (**C**–**E**) The organ coefficients of the liver, spleen, and kidney. Different alphabetical symbols signify statistical significance between different groups (*p* < 0.05).

The probiotic pretreatment experimental design is shown in Figure 1A. To investigate whether *L. plantarum* P101 pretreatment had existing effects on the body weight after the CTX injection, the body weight of mice for seven consecutive days post-CTX intervention was monitored. Results revealed that CTX exposure led to a significant decline in body weight, while the probiotic pretreatment exhibited a slight ameliorative effect. Nonetheless, no statistically significant difference was observed. In contrast, the body weight in the control group increased (Figure 1B). Additionally, the results indicated that CTX exposure significantly impacted the liver and spleen coefficients relative to the control group. Moreover, the liver coefficients of the probiotic pretreatment were higher in the CTX group, especially in the CTX + P101 (3 w) group (Figure 1C–E).

### 2.2. L. plantarum P101 Pretreatment Attenuated the CTX-Induced Liver Injury and Inflammation in Mice

**Figure 2:**
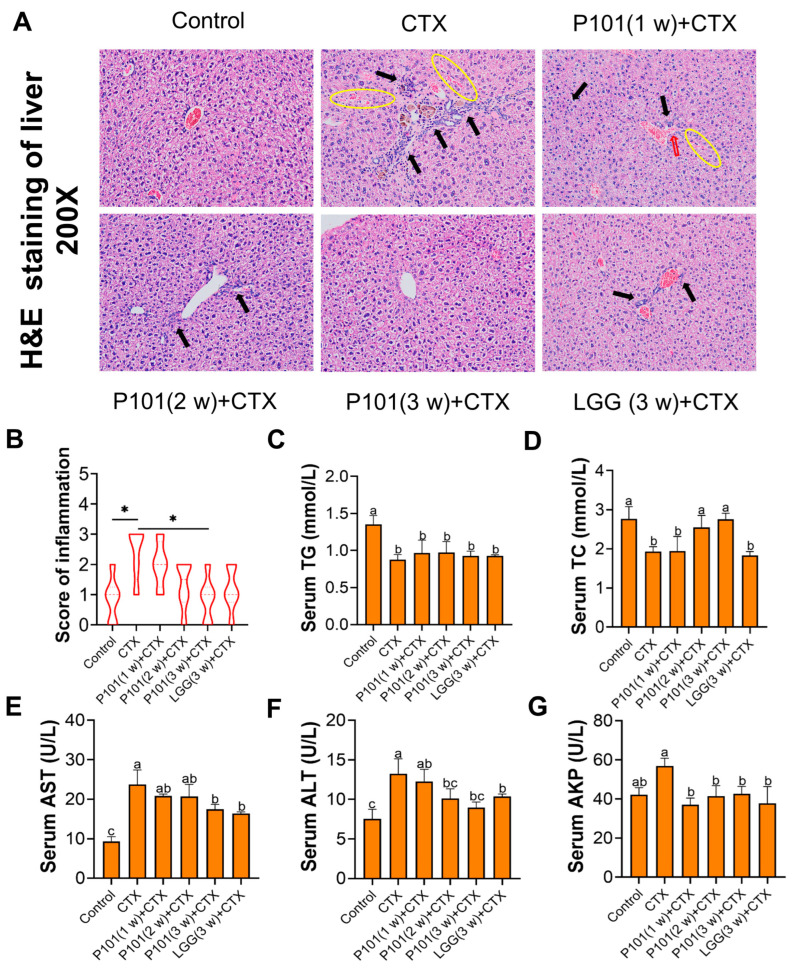
Effects of *L. plantarum* P101 pretreatment on CTX-induced liver injury in mice. (**A**) H&E staining images of the liver tissue (×200), hepatic blood sinuses were marked by a yellow cycle, and inflammatory infiltrates were marked by a black arrowhead. A blood vessel rupture was marked by a red arrowhead. (**B**) The score of inflammation. (**C**,**D**) The concentration of TG and TC in serum. (**E**–**G**) The AST, ALT, and AKP levels in liver tissue. Different alphabetical symbols signify statistical significance between different groups (*p* < 0.05). * *p* < 0.05 versus the specific two groups.

As shown in Figure 2A, the histopathological injury and inflammatory infiltration of the liver were assessed using H&E staining in different experimental groups. The CTX group exhibited structural disorder with irregular central venous shape and hepatic vascular rupture, as well as severe inflammatory infiltration and hepatic sinusoids following CTX intervention. Additionally, the ameliorative effects of the CTX + LGG (3 w) group were similar to those of the CTX + P101 (2 w) group but not as effective as the CTX + P101 (3 w) group. The score of inflammatory infiltration was consistent with the hepatic histopathology observation (Figure 2B).

To evaluate the preventive effect of probiotics on CTX hepatotoxicity, the concentration of TG, TC, AST, ALT, and AKP in serum was measured. The results revealed a noteworthy reduction in the contents of TG and TC in the liver after CTX intervention, and merely the TC level of the CTX + P101 (2 w) and CTX + P101 (3 w) groups was similar to those of the control group with no significant difference (Figure 2C,D). As illustrated in Figure 2E–G, the serum levels of AST, ALT, and AKP after 7 days of CTX exposure in the CTX group exceeded those of the control group. However, the mentioned indicators in the CTX + P101 (3 w) and CTX + LGG (3 w) groups were significantly lower than those of the CTX group and comparable to the control group. Moreover, the protective effect of the CTX + P101 (3 w) group was superior to that of the CTX + P101 (1 w) or CTX + P101 (2 w) groups, demonstrating a time-dependent ameliorative effect.

**Figure 3:**
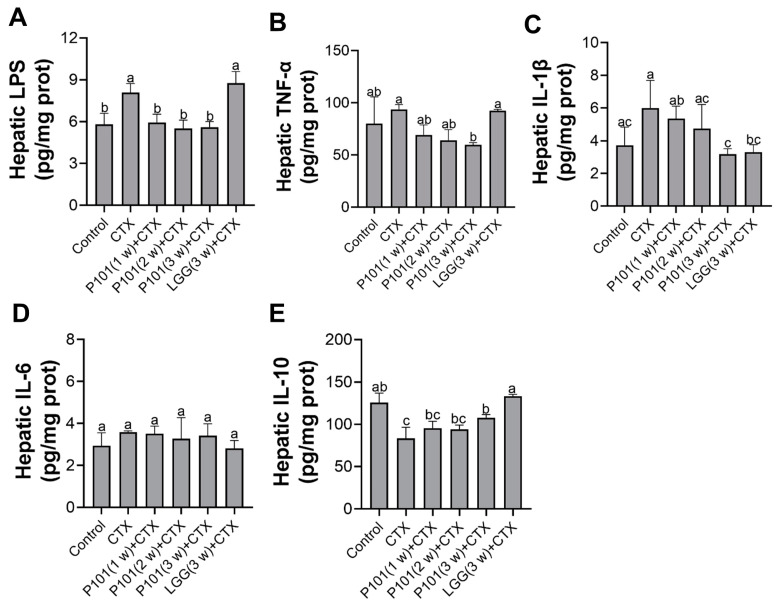
Effects of *L. plantarum* P101 pretreatment on the CTX-induced inflammatory responses of the liver in mice. (**A**) The concentration of LPS in liver tissue. (**B**–**E**) The level of inflammatory cytokines TNF-α, IL-1β, IL-6, and IL-10 in liver tissue. Statistical significance among groups (*p* < 0.05) was signified through different alphabetical symbols.

To further demonstrate hepatic injury and inflammation, the content of certain inflammation cytokines in liver tissue was assessed by ELISA. Results have found a significant increase in LPS in the CTX group compared to the normal group. However, in the CTX + P101 (1 w, 2 w, and 3 w) groups, probiotic administration reversed this trend and went back to the normal level, similarly to the control group. A slight increase in the content of TNF-α and IL-1β was observed after CTX exposure compared to normal levels. Interestingly, the pretreatments with *L. plantarum* P101 for three weeks minimized the content of those cytokines, whereas the CTX + LGG (3 w) group maintained comparable levels of those mentioned cytokines (TNF-α and IL-1β) to the CTX group (Figure 3A–C). In addition, the level of IL-6 was found to be similar across all groups, with no marked differences. When compared to the control group, a significant decrease in the content of IL-10 was found in the CTX group. Notably, only in the CTX + P101 (3 w) and the CTX + LGG (3 w) groups were the levels of IL-10 normalized back to those observed in the control group.

### 2.3. L. plantarum P101 Pretreatment Relieved CTX-Induced Oxidative Stress in the Liver

**Figure 4:**
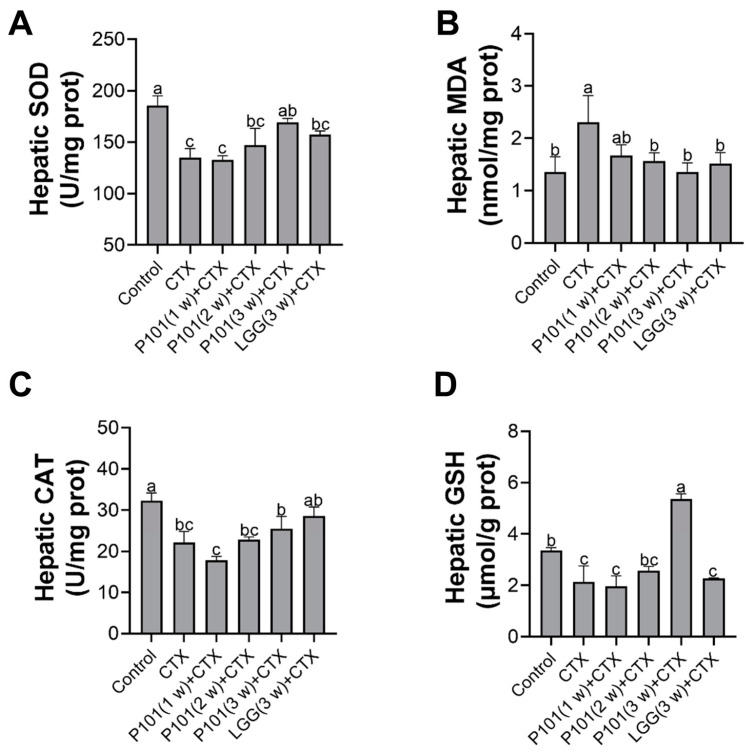
Effects of *L. plantarum* P101 pretreatment on the CTX-induced oxidative stress of the liver in mice. The contents of (**A**) SOD, (**B**) MDA, (**C**) CAT, and (**D**) GSH in liver tissue. Statistical significance among groups (*p* < 0.05) was signified through different alphabetical symbols.

The changes in activities of oxidases can serve as an indicator of oxidative stress in liver tissue, which was assessed in the current study. As depicted in Figure 4A, exposure to CTX results in a significant elevation of MDA, indicative of increased oxidative stress, whereas pretreatments with the probiotic groups did not yield any significant differences. Furthermore, the CTX treatment also exhibited a significant inhabitation in the activity of SOD and CAT enzymes in the liver and simultaneously decreased the concentration of GSH. The findings suggested that pretreatment with the probiotics for less than two weeks failed to provide significant improvements comparable to those in the CTX group. Only the probiotic pretreatment for three weeks demonstrated an effective reversal of the declining trend of the content of CAT, GSH, and SOD in liver tissue. Furthermore, the CTX + P101 (3 w) group exhibited superior effects as compared to the CTX + LGG (3 w) group (Figure 4B–D).

### 2.4. L. plantarum P101 Pretreatment Activated the Nrf2/ARE Signaling Pathways in the Liver

**Figure 5:**
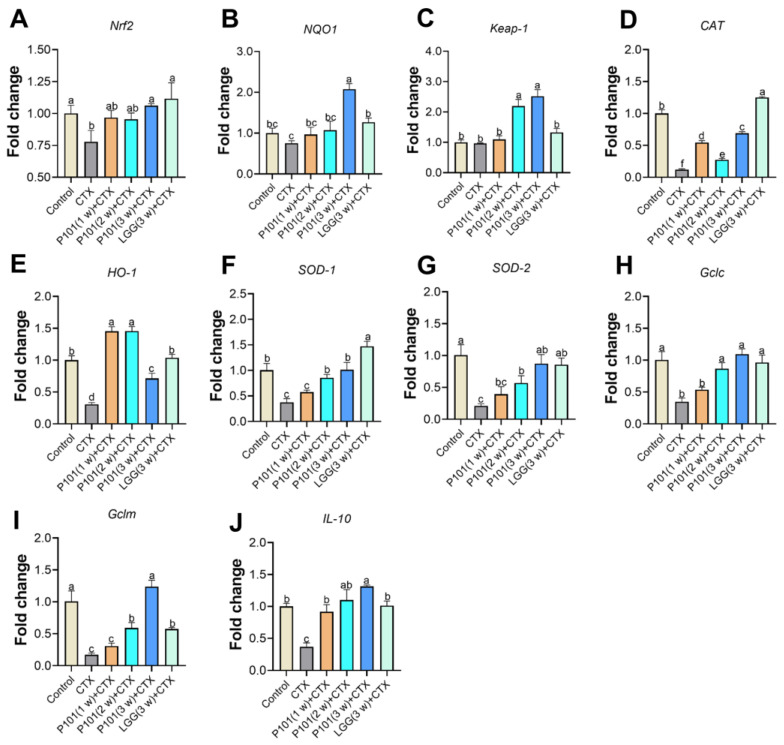
Effects of *L. plantarum* P101 pretreatment on the Nrf2/ARE signaling pathways of the liver in mice. The mRNA expression of (**A**) *Nrf2*, (**B**) *NQO1*, (**C**) *Keap-1*, (**D**) *CAT*, (**E**) *HO-1*, (**F**) *SOD-1*, (**G**) *SOD-2*, (**H**) *Gclc*, (**I**) *Gclm*, and (**J**) *IL-10*. Statistical significance among groups *(p* < 0.05) was signified through different alphabetical symbols.

**Figure 6:**
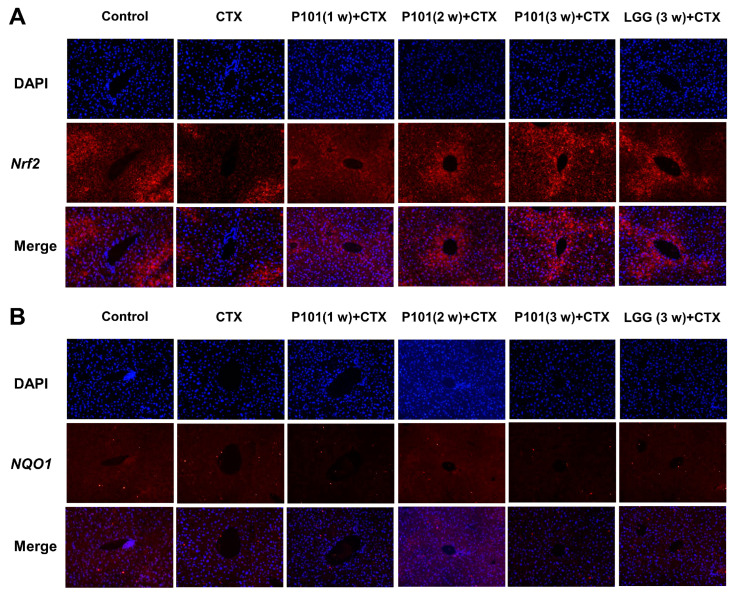
Immunofluorescence detection of Nrf2 and NQO1 in the liver in mice. (**A**) Nrf2; (**B**). NQO1. Blue: DAPI; red: Nrf2; and NQO1, magnification: 200×.

To investigate the effects of *L. plantarum* P101 pretreatment on the *Nrf2* signaling pathway, the mRNA expression of relevant genes involving the signaling pathway was analyzed. The results revealed that the mRNA expression levels of *Nrf2* and its downstream genes, such as *NQO1*, *CAT*, *HO-1*, *SOD-1*, *SOD-2*, *Gclc*, and *Gclm*, in the CTX group had noticeably decreased compared to those of the control group. However, most of the downregulated genes were restored and upregulated in the probiotic pretreatment groups, and the 3 w (long-dose) group showed a great elevation in the mRNA levels compared to the short-dose group. Results showed that antioxidative genes that decreased after the CTX intervention had a significant elevation in the CTX + P101 (3 w) and the CTX + LGG (3 w) groups, and both pretreatments exhibited similar abilities in restoring the mRNA expression levels related to the *Nrf2* signaling pathway (Figure 5A–I). The expression level of *IL-10* also showed a similar trend to the forementioned genes (Figure 5J). Furthermore, the immunofluorescence analysis results show that Nrf2 protein levels were consistent with gene expression levels. Whereas no significant difference was observed between the groups in terms of NQO1 protein expression (Figure 6A,B).

### 2.5. L. plantarum P101 Pretreatment Alleviated CTX-Induced Hepatocytes Apoptosis in the Liver

**Figure 7:**
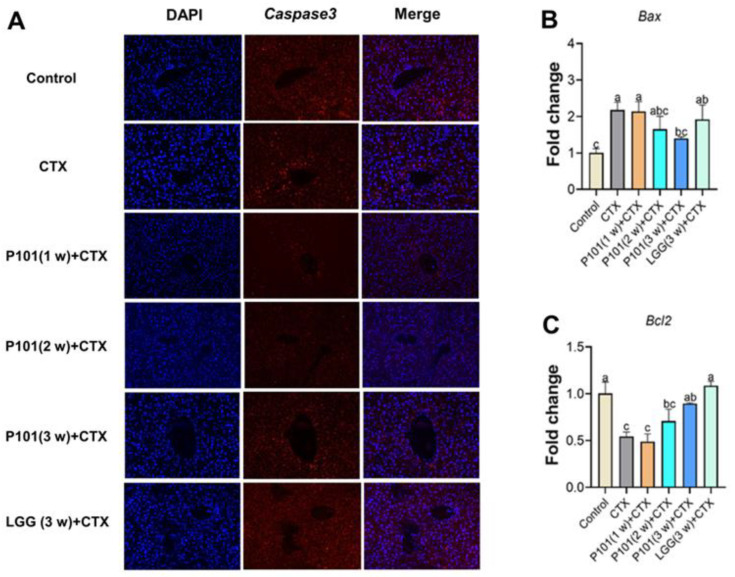
Effects of *L. plantarum* P101 pretreatment on the expression of apoptosis-related genes and liver proteins in mice. (**A**) Caspase3 immunofluorescence (IF) staining of the liver in mice. (**B**,**C**) The mRNA expression of *Bax* and *Bcl2*. Different alphabetical symbols signify significant differences (*p* < 0.05) among groups.

To further verify the effects of probiotic pretreatment on the hepatotoxicity induced by CTX, hepatocyte apoptosis was analyzed. The expression level of Caspase-3 in the CTX group was significantly higher than that in the control group. However, the combined use of probiotic pretreatment with CTX intervention reduced the increasing levels of Caspase-3. Moreover, the Caspase-3 level of the CTX + P101 (3 w) group was lower than that of the CTX + P101 (1 w, 2 w) groups but higher than that of the CTX + LGG (3 w) group (Figure 7A). Furthermore, the mRNA expression of *Bax* and *Bcl2* was also measured, two important genes involved in apoptosis regulation. Results have suggested that the mRNA expression of *Bax* was significantly increased in the CTX group compared with the control group, while *Bcl2* markedly decreased (Figure 7B,C). Additionally, the *L. plantarum* P101 pretreatment had time-dependent ameliorative effects against CTX-induced hepatocyte apoptosis. Additionally, the CTX + P101 (3 w) group exhibited the best comprehensive effect, which was equivalent to that of the CTX + LGG (3 w) group.

## 3. Discussion

Cyclophosphamide (CTX) is a widely used anti-tumor and immunosuppressant, but its adverse effects have imposed significant burdens and pains on cancer patients worldwide [26,27]. Therefore, it is imperative and urgent to explore alternative treatments that can reduce or even eliminate adverse reactions to CTX during its treatment. Accumulating studies have reported that *Lactobacillus plantarum* exhibits considerable beneficial effects in various liver disease models, including alcoholic liver disease [22], as well as D-galactosamine and lipopolysaccharide-induced liver injury [28,29]. Moreover, existing studies reported that CTX-induced hepatotoxicity is likely to be associated with the oxidative stress caused by excessive ROS, thereby eliminating the excessive ROS may be a potential and feasible strategy to reduce CTX cytotoxicity [30]. Probiotics are known for their antioxidant capacity, and the use of different probiotics targeting one or more diseases may exert varying effects. Therefore, it is important to investigate the underlying mechanism and the optimal intervention period for the use of different probiotics to treat liver injury.

After administering CTX to mice at a dose of 30 mg/kg body weight for 7 days, chronic and low-dose environmental exposure resulted in a declining trend between the body weight and organ coefficients, indicating the presence of significant tissue damage to the liver and spleen in mice, consistent with previous research [31,32]. Additionally, the administration of CTX led to hepatotoxicity, as evidenced by an increase in liver function damage indicators (AKP, AST, and ALT) in serum [33,34,35]. These indicators are strongly associated with the necrosis, degeneration, and inflammatory response of hepatocytes [8], which will leak from the cytoplasm and traverse the damaged cytomembrane into the blood circulation. The results of this study confirmed the emergence of severe inflammatory infiltration and destruction in the liver following CTX exposure. The pretreatment of *L. plantarum* P101 and LGG effectively alleviated the hepatotoxicity induced by CTX and reversed the abnormal changes in the liver. The treatment effect was found to be time- and dose-dependent. Similarly, some medicinal plants have been shown to ameliorate CTX-induced hepatotoxicity via their antioxidant activities [36,37,38], indicating the anti-inflammatory and antioxidative activities of Lactobacillus plantarum may also be crucial factors for those amelioration effects of probiotics. However, the underlying connection between the duration of prevention and the degree of protection is still unclear. Interestingly, there were abnormal changes in TG and TC content in mice treated with CTX. It is speculated that it may be attributed to acrolein binding to lipids and reacting, interfering with the lipid metabolism system in the liver, but the specific mechanism needs further analysis and exploration.

The inflammatory response is closely associated with the development of CTX-induced hepatotoxicity. Once liver tissue is injured, it can cause the secretion and release of pro-inflammatory cytokines by immune cells and damaged cells [33,38]. NF-κB signaling is a major signaling pathway involved in gene regulation and activation of pro-inflammatory cytokines, including iNOS, IL-1β, and TNF-α [5,39]. A study reported that CTX could activate NF-κB through ROS and subsequently upregulate the expression of various inflammatory factors [5]. Li et al. [4] found that pyrroloquinoline quinone treatment significantly inhibited the increased levels of IL-1β, IL-6, and TNF-α in the liver induced by CTX. Additionally, the results have demonstrated that prophylaxis with *L. plantarum* P101 effectively blocked overproduction of inflammatory cytokines (LPS, TNF-α, and IL-1β) to exert anti-inflammatory and protective activity. It has been demonstrated that CTX could disrupt intestinal immunity and the integrity of the intestinal barrier. That can lead to more LPS produced by harmful bacteria entering the liver through the disrupted intestinal and portal veins, which may further activate Kupffer cells and promote the production and secretion of inflammatory cytokines [40]. Intestinal microorganisms are closely related to human health and the pathogenesis of liver disease [41]. Here, the distal organ protective effect of *L. plantarum* P101 may be achieved by antagonizing and inhibiting the growth of harmful bacteria in the intestinal tract. In addition, the 3 week intervention with *L. plantarum* P101 has a better regulation of the inflammatory response than the shorter intervention, which may account for the intestinal flora, but further research is needed. Meanwhile, the LGG group maintained higher levels of LPS and TNF-α than those of the *L. plantarum* P101 group with the same intervention period. That also indicates that probiotics exhibit a better curative effect in specific fields rather than an overall advantage in all fields. All in all, *L. plantarum* P101 can effectively alleviate CTX-induced inflammation and perform better protective effects in some respects than LGG.

Oxidative stress has been linked to CTX-induced hepatic injury. The active metabolite of CTX, acrolein, activates xanthine oxidase, which enhances ROS production and causes induced oxidation reactions [8]. A previous study showed that rats receiving CTX (30 mg/kg/day) via intraperitoneal injection for 10 consecutive days had elevated lipid peroxidation and decreased antioxidant defense [26]. MDA, a marker of lipid peroxidation, significantly increased after CTX exposure in this study, indicating an elevated level of ROS in mice. Instead, the SOD enzyme, which is a key ROS scavenger [42], is involved; hence, the changes in the mentioned MDA and SOD activity from those results indirectly reflect the emergence of oxidative stress. Protecting hepatocytes from oxidative damage and reducing the free radical formation induced by acrolein, GSH is important and crucial. As a result, GSH depletion leads to the decline of the cellular defense against free radical-induced damage, eventually resulting in inflammation and cell necrosis [33,43]. The results indicated that pretreatment with *L. plantarum* P101 for three weeks effectively reversed the oxidative stress induced by CTX, whereas short-term treatment had insignificant effects. That suggests that exogenous probiotics require sufficient time to adapt to the host intestinal environment to exert beneficial effects. In other words, it also implies that supplementation with *L. plantarum* P101 requires at least three weeks to exert good protective effects on the liver. Previous studies have reported that high-concentration probiotic supplementation is more effective than low-concentration probiotics in alleviating CTX-induced intestinal immune damage and constipation [44,45,46]. Generally, these results suggest that the administration for three weeks of *L. plantarum* P101 is an effective method for alleviating CTX-induced liver oxidative stress.

To further elucidate the molecular antioxidant mechanism involved in *L. plantarum* P101 and LGG against CTX-induced hepatic injury, the Nrf2/ARE pathway and its downstream antioxidant gene expression were evaluated. Upon exposure to external stimuli, such as oxidative stress, Nrf2 will be released from the Nrf2-Keap1 compound existing in the cytoplasm and enter the nucleus, thus regulating the transcriptional expression of genes related to downstream antioxidant enzymes [5,47]. It has been reported that CTX administration could notably inhibit the transcriptional expression of Nrf2 and its downstream genes (*NQO1*, *HO-1*, and *Gclm*) in the liver [4,48]. In this study, CTX treatment markedly suppressed the pathway of Nrf2/ARE and indeed downregulated the expression of downstream target genes, including *HO-1*, *CAT*, *SOD-1*, *SOD-2*, *Gclc,* and *Gclm*, consistent with biochemical data. These findings indicated that the depletion of the antioxidant defense system in CTX-induced hepatoxicity is necessary and involves the regulator of the Nrf2/ARE pathway. That may be caused by acrolein triggering the generation of intracellular ROS and disturbing the antioxidant mechanism [49]. Meanwhile, some reports have demonstrated that the protective effect of probiotics in various disease models is related to the activation of the Nrf2 pathway [50,51,52]. Collectively, our results suggest that probiotics can reduce CTX-induced hepatotoxicity in vivo by activating the Nrf2/ARE pathway, with a longer prevention duration showing greater efficacy. Moreover, other molecular mechanisms or pathways involving the hepatoprotective effect of *L. plantarum* P101 and LGG need to be further studied.

In addition to inflammation and oxidative stress, CTX-induced hepatocellular toxicity is mediated by apoptotic responses [6]. Apoptosis is an initial cell death process controlled by multiple genes [53] and triggered by various stimuli, including oxidative stress and inflammation responses. Liu et al. [49] found that acrolein activated the mitochondrial apoptosis pathway by regulating the ratio of *Bcl2/Bax*. Bcl2 acts as an anti-apoptotic mechanism for various stimulations, but Bax can speed up apoptosis. Similarly, allicin can protect the liver from CTX hepatoxicity by decreasing the ratio of *Bax/Bcl2* and the protein level of Caspase 3 [48]. In addition, Caspase 3 is a reliable indicator of the severity of apoptosis [53]. Additionally, *L. plantarum* P101 and LGG could markedly alleviate the hepatocyte apoptosis induced by CTX, which may be explained by their excellent anti-inflammatory and antioxidant properties. It seems that only using *L. plantarum* P101 and pretreating the mice for three weeks can exhibit effective inhibition of hepatocyte apoptosis. Additionally, whether LGG needs the longer period needs to be further studied. All in all, the study evaluated the different strains (*L. plantarum* P101 and LGG) and intervention duration on the protective effects of CTX-induced liver injury and provided a potential therapeutic strategy and new information.

## 4. Materials and Methods

### 4.1. Probiotics and Reagents

The strain of *L. plantarum* P101 (CCTCC M 2021108) used in this work was separated from traditional Chinese preserved foods (pickled cabbage). Man–Rogosa–Sharpe (MRS) and Cyclophosphamide (CTX) were both obtained from Beijing solarbio science and technology Co., Ltd. (Beijing, China). 1% phosphate buffer (1 × PBS, pH 7.2, 0.01 M) consisted of sodium chloride (NaCl), potassium chloride (KCl), and potassium dihydrogen phosphate (KH_2_PO_4_).

### 4.2. Preparation of Bacterial Strains

The two strains (*L. plantarum* P101 and LGG) were cultured at 37 °C in sterile MRS for 16 h in an anaerobic environment. The bacterial mixtures were subjected to high-speed centrifugation at 12,000 rpm for 2 min to preserve the precipitates, which were resuspended twice with 1 × PBS to adjust the bacterial concentration to 10^8^ CFU/100 µL according to our previous methods [18].

### 4.3. Experimental Design

Male C57BL/6 mice (weight = 24.0 to 26.0 g) were obtained from SLAC Jingda Laboratory Animal Company (Changsha, Hunan, China). These mice were fed in cages in a specific sterile environment with constant ambient humidity and a temperature of 24 ± 1 °C, alternating between day and night every 12 h, and were allowed to drink and eat freely. After a week of adaptation, mice were assigned randomly to six groups (*n* = 8): the control group, the Cyclophosphamide-treated group (CTX), the CTX-treated with P101 group with three different durations of oral gavage pretreatment (1 week, 2 weeks, and 3 weeks), and the CTX-treated with LGG group with a three-week period of oral gavage pretreatment (CTX + LGG (3 w)) as a positive control group. The normal control group was administered sterile saline via intraperitoneal injection, while the other groups were exposed to 30 mg/kg BW CTX by intraperitoneal administration once daily for 7 consecutive days during the last week. The normal control group and CTX group were treated with sterile saline via gavage for the first three weeks in a row. While CTX + P101 (1 w), CTX + P101 (2 w), CTX + P101 (3 w), and CTX + LGG (3 w) were administered with three different durations of oral gavage pretreatment (2 weeks, 1 week, and 0 week), and oral gavage of bacterial solution (10^8^ CFU) once a day for the rest of the time until modeling. All mice were sacrificed on the 29th, and some necessary tissue samples were stored for further study.

### 4.4. Histopathological Analysis and Serum Biochemical Analysis

Fresh samples of liver tissue were immersed in a 4% paraformaldehyde solution, followed by dehydration through an ethanol gradient and xylene and paraffin-embedded tissue samples. Subsequently, the tissue was sliced into 5 μm-thick sections and finally stained with hematoxylin-eosin (H&E) under an inverted fluorescence microscope (Japan, Tokyo) to observe its pathological structural damage and obtain representative pictures. The histological score was assessed concerning the degree of inflammatory infiltration following the criteria described by Zhao et al. [54]. Those indicators revealing the health of the liver were analyzed by commercial kits purchased from Nanjing jiancheng Bio-tech Co., Ltd. (Nanjing, China). Those kits contain ranges for aspartate aminotransferase (AST), alanine aminotransferase (ALT), alkaline phosphokinase (AKP), total cholesterol (TC), and triglyceride (TG), and all protocols strictly comply with its instructions and guide.

### 4.5. Determination of Oxidative Stress in the Liver

The liver tissue sample was weighted, and sterile normal saline was added at a 1:9 ratio to prepare the 10% (*w*/*v*) homogenate, which was then centrifuged at 3000× *g* for 15 min at 4 °C to retain the supernatant for analysis. The BCA protein assay kit (Applygen Technologies Inc.) was utilized to detect its albumen concentration. The activities of superoxide dismutase (SOD) and catalase (CAT), malondialdehyde (MDA) level, and reduced glutathione (GSH) content in the prepared homogenate were analyzed by commercial kits, which were bought from Nanjing jiancheng Bio-tech Co., Ltd. (Nanjing, China).

### 4.6. Determination of Liver Inflammatory Factors

Following the BCA method, the protein concentration of 10% liver homogenate supernatant was quantified. Subsequently, enzyme-linked immunosorbent assay (ELISA) kits (Shanghai YSRIBIO Industrial Co., Shanghai, China) were utilized for evaluation of the expression content of interleukin (IL-6), IL-10, IL-1β, tumor necrosis factor -α (TNF-α), and lipopolysaccharide (LPS) in mice. Additionally, all procedures were conducted in accordance with the instructions in the kits.

### 4.7. Real-Time Quantitative PCR (RT-qPCR) Analysis

Fresh liver tissue was processed using the AxyPrep Multisource Total RNA Miniprep Kit (Axygen Scientific, San Francisco, CA, USA), and the guidelines were followed to extract the total RNA. Then, using the NanoDrop 1000 spectrophotometer (Thermo Scientific Inc., San Francisco, CA, USA) to quantify the content of RNA, it was adjusted prior to cDNA inversion. The procedures for cDNA inversion were conducted in accordance with the Takara PrimeScript TM RT reagent (Cat#RR047A, Lot#AK2802) kit (Takara Bio Inc., Ostu, Japan). For RT-qPCR reactions, it involves the participation of the TB Green^®^ Premix Ex Taq™ II kit (Takara Bio Inc., Ostu, Japan) and the AriaMx Real-time qPCR system from Agilent (lot number MY 19435252, Agilent Technologies, Santa Clara, CA, USA). Relative gene expression was detected by using the threshold cycling method (2^−ΔΔCt^), and its internal reference gene was selected for β-actin gene. Primer information can be found in Table S1.

### 4.8. Immunofluorescence Assay

As for the deparaffinization of liver tissue paraffin sections, xylene and ethanol reagents were utilized for this process. Then antigen retrieval was performed using EDTA buffer (pH 8.0). Blocking was performed using 3% BSA serum for half an hour. Then it was incubated with primary antibodies (Caspase3, Nrf2, and NQO1) overnight at 4 °C. Subsequently, the sections were covered with CY3 goat anti-rabbit fluorescent secondary antibody (Servicebio, GB21303, Wuhan, China) and incubated for 50 min without light. DAPI re-stained the nuclei, quenched the tissue for self-fluorescence, and sealed the slices. Finally, using fluorescence microscopy (Nikon Eclipse Ci) to visualize the slices, immunofluorescence pictures were acquired.

### 4.9. Statistical Analysis

The group-wise comparisons were performed using one-way analysis of variance (Tukey test) in GraphPad Prism 8.0.1 software (San Diego, CA, USA), with the data presentation as means and standard deviations. When *p* < 0.05 was set as statistical significance existing in groups.

## 5. Conclusions

In summary, the potential effects of two strains (*L. plantarum* P101 and LGG) on CTX-induced hepatotoxicity were evaluated in mice. The results showed that CTX indeed induced severe injury in the liver, and the *L. plantarum* P101 pretreatments showed better comprehensive effects in attenuating CTX-induced liver injury via inhibiting oxidative stress and inflammation than LGG pretreatments, and the longer duration of prevention had better protection than the shorter duration in the present results. Furthermore, it was also confirmed that the beneficial promoting effects of two probiotics are associated with regulation of the Nrf2/ARE signaling pathway in the liver in mice and provide a potential therapeutic strategy against CTX-induced liver injury.

## Data Availability

The data used to support the findings of this study can be made available by the corresponding author upon request.

## Supplementary Materials

The following supporting information can be downloaded at: https://www.mdpi.com/article/10.3390/ijms241713424/s1.
