# Supplementary material for: Lactiplantibacillus plantarum P101 Attenuated Cyclophosphamide-Induced Liver Injury in Mice by Regulating the Nrf2/ARE Signaling Pathway
Source: Int J Mol Sci. 2023 Aug 30;24(17):13424. doi: 10.3390/ijms241713424 (PMC10488115; doi:10.3390/ijms241713424)
Supplement: Supplementary file 1 [file ijms-24-13424-s001.zip › ijms-2571939-supplementary.pdf]

Supplementary material

Lactiplantibacillus plantarum P101 attenuated cyclophosphamide-induced liver injury in mice by regulating Nrf2/ARE signaling pathway

*Tao You, Yu Zhao, Shanji Liu, Hengyi Xu**

State Key Laboratory of Food Science and Resources, Nanchang University, Nanchang 330047, China.

*Correspondence to:

Dr. Hengyi Xu

State Key Laboratory of Food Science and Resources, Nanchang University.

Address: 235 Nanjing East Road, Nanchang 330047, P. R. China.

Phone: +0086-791-8830-4447-ext-9520.

Fax: +0086-791-8830-4400.

E-mail: kidyxu@163.com, HengyiXu@ncu.edu.cn.

Table S1. List of genes and primers used in this study.

Gene	Primer	Sequence (5’→3’)
Nrf2	Forward	ACTTCGGTGATTCTGTTAG
	Reverse	CTCGTGCTCGCTTACT
NQO1	Forward	AGGATGGGAGGTACTCGAATC
	Reverse	TGCTAGAGATGACTCGGAAGG
Keap-1	Forward	GAAGAGGCGGCAGAAGAAG
	Reverse	GCTCCAGGGCTATGACAGAT
HO-1	Forward	ACCGCCTTCCTGCTCAAC
	Reverse	GAGGAGCGGTGTCTGGGAT
CAT	Forward	TTGTTCAGTGACCGAGGGATT
	Reverse	TTCCTGAGCAAGCCTTCCTG
SOD-1	Forward	TAACTGAAGGCCAGCATGGGT
	Reverse	GGTCTCCAACATGCCTCTCTTC
SOD-2	Forward	CAGACCTGCCTTACGACTATGG
	Reverse	GCTGAAGAGCGACCTGAGTTGT
Gclc	Forward	ATGTGGACACCCGATGCAGTATT
	Reverse	TGTCTTGCTTGTAGTCAGGATGGTTT
Gclm	Forward	CTTCGCCTCCGATTGAAGATG
	Reverse	AAAGGCAGTCAAATCTGGTGG
IL-10	Forward	TAACTGCACCCACTTCCCAG
	Reverse	AAGGCTTGGCAACCCAAGTA
Bax	Forward	GATGGCAACTTCAACTGGG
	Reverse	CCGAAGTAGGAGAGGAGGC
Bcl2	Forward	CACTCGACCTTGTTTCTTCCAG
	Reverse	TCCTAACCCCTTGCTCTGCTT
β-actin	Forward	GCTCCTCCTGAGCGCAAGTA
	Reverse	CAGCTCAGTAACAGTCCGCC
